# Inhibition of the Constructive Aggression Behavioral System (CABS) is associated with poor short- and long-term treatment outcomes and readmission among patients with major depression: a naturalistic study

**DOI:** 10.1186/s12888-026-08490-3

**Published:** 2026-09-11

**Authors:** Reinhard Maß, Kerstin Backhaus, Kevin Pütz, Michael Szelies

**Affiliations:** 1 Institute for Psychology, University of Hamburg, Von-Melle-Park 5, 20146 Hamburg, Germany; 2 Center for Mental Health Marienheide, Marienheide, Germany

**Keywords:** Constructive Aggression Behavioral System, CABS, Transdiagnostic approach, Evolutionary psychology, Emotional disorders, Major depressive disorder, Psychotherapy outcome, Prognosis, Psychometric measures

## Abstract

**Background:**

The Constructive Aggression Behavioral System (CABS) has been conceptualized as an evolutionarily grounded pattern of behavior that enables individuals to assert their needs, interests, and boundaries in a non-violent and socially adaptive manner. It is theoretically distinct from destructive forms of aggression. Inhibition of CABS has been proposed as a clinically relevant construct in emotional disorders. The present study aimed to examine the role of CABS inhibition in treatment outcomes and longer-term prognosis in patients with major depressive disorder (MDD).

**Methods:**

A total of 416 inpatients diagnosed with MDD were assessed using the Aggressionshemmungs-Inventar (AHI) to measure CABS inhibition and the revised Beck Depression Inventory (BDI-II) at admission and discharge. Three hierarchical multiple regression analyses were conducted to examine whether AHI scores at discharge were associated with change in depressive symptoms from admission to discharge and whether they predicted depressive symptoms at 6-month follow-up as well as psychiatric readmission within one year. Covariates included age, gender, suicidality, unemployment, comorbid anxiety, and antidepressant use.

**Results:**

At admission, patients showed elevated AHI scores (mean z = 1.40), indicating substantial inhibition of constructive aggression. During treatment, both depressive symptoms and AHI scores decreased significantly. AHI scores at discharge were associated with symptom change during treatment and with depressive symptoms at 6-month follow-up as well as readmission within one year.

**Conclusions:**

The findings indicate that CABS inhibition represents a clinically relevant correlate of treatment outcome and is associated with follow-up outcomes in MDD. Given the observational design, the results do not allow conclusions regarding causal effects. Future longitudinal and experimental studies are needed to examine whether changes in CABS inhibition are associated with changes in clinical outcomes and to further clarify its potential role in the onset and maintenance of emotional disorders.

**Clinical trial number:**

Not applicable.

## Introduction

Aggression is traditionally understood as deliberate, harmful behavior directed toward others, with the intent to inflict physical or psychological damage. A definition from a standard reference work is as follows: “Aggression is any form of behavior directed toward the goal of harming or injuring another living being who is motivated to avoid such treatment” ([2], p. 7). In the following text, we refer to this type of behavior as *destructive aggression*. From an evolutionary psychology perspective, the primary domain of destructive aggression lies in the competition *between rival social systems*. One example of this is the chimpanzee wars described by Jane Goodall, which can lead to the physical annihilation of an entire group [23]. There is also evidence for this in humans, dating back to the Bronze Age [51]. Destructive aggressive behavior serves to address various adaptive problems, such as acquiring resources from others, exerting power, and deterring rivals [9].

At the same time, there is compelling evidence that certain fundamental aspects of aggression may be beneficial—and even essential—for both individual health and overall quality of life:

1. In contemporary emotion theories, anger is understood as the primary emotional trigger of aggressive behavior (e.g., the RAGE/ANGER emotion system; [40]). *Suppression of anger* has been associated with various adverse outcomes, including social anxiety and perceived stress [56], reduced job satisfaction [6], depressive symptoms [43], and elevated blood pressure [41].
2. Although aggression is conceptually distinct from assertiveness, assertiveness approaches commonly involve behavioral elements that closely resemble those found in aggression—such as a willingness to engage in conflict, to enforce personal boundaries, or to oppose the interests of others when necessary [1].
3. Evidence from game-theoretic research [17] suggests that altruistic punishment—the willingness to punish unfair behavior despite incurring personal costs—is often driven by anger in response to perceived injustice and may constitute a key psychological mechanism underlying the evolution of human cooperation. Socio-anthropological analyses [45] further indicate that egalitarian strategies aimed at preventing inequalities in power and income, such as altruistic punishment, provide the best explanation for observed behavioral patterns in a real hunter-gatherer society.
4. In the psychoanalytic literature [27], the term aggression is traditionally understood not only as destructive behavior but also as a form of “constructive force” that is important for personality development and social relationships.

We address this fundamental issue by proposing a distinct, evolutionarily shaped pattern of human behavior that is both functionally and conceptually separable from destructive aggression. We refer to this hypothesized pattern as the Constructive Aggression Behavioral System (CABS). We assume that, unlike destructive aggression, the CABS derives its adaptive value not from interactions *between* rival social systems, but from its role *within* social systems—such as romantic partnerships, families, and peer groups. Rather than aiming to harm others, CABS contributes to addressing a core adaptive challenge faced by all social systems: managing the trade-off between competing and cooperative interests among their members. A social system can only function effectively and serve the interests of all its members if this challenge is successfully resolved. Destructive aggression is poorly suited to this purpose, as it risks injuring or eliminating group members, thereby causing significant resource loss to the group as a whole. The CABS may play an essential role in resolving the trade-off problem described above by helping to balance cooperative and competitive interests [52], for example in contexts such as hunting [25] or sexual reproduction [19]. Mayr, [39] introduced the distinction between proximate causes—those that directly elicit behavior—and ultimate causes, which concern the adaptive function of behavior. In this framework, constructive aggression allows individuals to assert their interests, needs, and boundaries within the group (proximate cause). In doing so, the CABS may contribute to the stability and adaptive success of the social system (ultimate cause). Constructive aggression is nonviolent in nature and can be expressed both verbally and nonverbally—through facial expressions, posture, and tone of voice. The CABS is understood as a behavioral pattern rather than an emotional state. Nevertheless, emotions such as desire, anger, or fear may act as triggers for the CABS. Situational examples include the unjust distribution of essential resources (associated emotion: anger), the presence of an attractive person (desire), or a violation of personal boundaries (fear/anger). The function of the CABS response is, depending on the situation, to achieve just redistribution, a desired approach, or necessary distancing.

### Inhibition of the CABS

The CABS should be understood as an innate potential that is shaped and modified by psychological influences (e.g., upbringing, formative childhood experiences) as well as societal factors (e.g., social norms, religious dogmas). In some cases, these influences lead to the suppression of constructive aggression, a phenomenon referred to in this paper as *inhibited CABS*. This term denotes a temporally stable and cross-situational tendency to suppress healthy, natural, constructive-aggressive behavior caused by psychological and/or societal factors. Consequently, it is hypothesized that an inhibited CABS may impair an individual’s ability to adequately attend to their own needs, interests, and personal boundaries. This would, in turn, contribute to the development of various interpersonal difficulties and mental disorders.

### Previous studies on the CABS

Recently, a series of three studies on the CABS was presented by Maß and Müller-Alcazar [37]. The aim of Study 1 was to develop a new questionnaire to assess the inhibition of the CABS. A pool of 78 concise items was generated, addressing the following thematic domains: ethical or religious convictions that may inhibit the expression of constructive-aggressive impulses (e.g., strict pacifist beliefs such as “If anyone slaps you on the right cheek, turn to them the other also”); catastrophizing appraisals of interpersonal conflict; biographical constellations potentially conducive to dysfunctional core beliefs, such as formative experiences of violence, loss of control in childhood, or disproportionate punishment (e.g., emotional or social withdrawal) for undesirable behavior; potential health consequences of aggression inhibition, particularly in the form of psychosomatic stress symptoms; attitudes toward anger (e.g., acceptance vs. rejection); fear of losing control (e.g., engaging in violent behavior); experiencing guilt; being criticized by others; conflation of anger with violence; fear of others’ anger; conflict avoidance; excessive need for harmony and overcompliance; neglect of personal interests, needs, and boundaries; and frequent subjective experiences of helplessness or powerlessness. Factor analysis yielded a unidimensional 20-item scale termed the Aggressionshemmungs-Inventar (AHI; [38]). Example items include:

- “I’m good at standing up for my needs and interests.” (reversed)
- “When someone gets angry at me, it scares me.”
- “I have a hard time standing up for myself when someone crosses a line.”
- “If I want something, I just take it.” (reversed)

The degree of agreement was aggregated into a total score, which quantifies the extent to which the CABS is inhibited. Among other findings, in this nonclinical sample, depressive symptoms—as measured by the BDI-II [3]—were found to be highly significantly associated with the AHI (*r* = .54, *p* < .001).

Study 2 (*N* = 253 healthy adults) served to evaluate the findings of Study 1. In addition to the BDI-II (correlation with the AHI: *r* = .48, *p* < .001), the trait scale of the State-Trait Anxiety Inventory (STAI; [34]) was administered. This 20-item scale assesses chronic anxiety; its correlation with the AHI was *r* = .66 (*p* < .001). The participants also completed the “Fragebogen zur Lebenszufriedenheit” (FLZ; [15]), a questionnaire measuring quality of life. The FLZ total score reflects quality of life across seven life domains, each assessed with seven items: physical health, financial situation, leisure, self-perception, sexuality, social relationships (friends/acquaintances/relatives), and living conditions. Overall quality of life, as assessed by the FLZ, was also significantly associated with the AHI score (*r* = − .50, *p* < .001).

Study 3 aimed to examine the applicability of the AHI, as developed in Studies 1 and 2 with healthy adults, in a clinical sample. A total of 263 inpatients diagnosed with major depressive disorder (MDD) were assessed. This sample presented markedly elevated AHI scores compared with those of healthy controls. When converted into standardized *z*-scores on the basis of the distribution parameters of the healthy control samples from Studies 1 and 2, the depressed patients obtained a mean value of 1.35. Furthermore, a significant association between depressive symptoms (BDI-II) and the AHI score was again observed in this clinical sample (*r* = .373, *p* < .001). Altogether, Study 3 demonstrated that the AHI is well-suited for use in clinical research.

## Objectives and hypotheses

The present study investigated the clinical relevance of the CABS model in the treatment of inpatients with MDD. We hypothesized that (1) patients with MDD would be characterized by CABS inhibition, (2) this inhibition would be reduced during psychotherapy, and (3) the level of CABS inhibition at discharge would be associated with symptom reduction from admission to discharge, depressive symptom severity at six-month follow-up, and readmissions within one year after discharge.

## Methods

### Sample characteristics

A total of 416 inpatients consecutively admitted to the “Aaron T. Beck” ward at the Center for Mental Health Marienheide, Germany, with a diagnosis of MDD, were included in the study. (The patients from Study 3—see above—are part of the current sample.) Recruitment period: the first patient was enrolled in October 2018, and the last patient was discharged in September 2024. The final 6-month follow-up was completed in March 2025, and the one-year observation period concluded in September 2025. Diagnostic criteria according to the ICD-10 were assessed using the International Diagnostic Checklists (IDCL; [30]). The sample comprised 172 men (41.3%) and 244 women (58.7%), with a mean age of 40.9 years (SD = 14.1; range = 18–73). Exclusion criteria included comorbid personality disorders, obsessive–compulsive disorder, psychotic disorders, bipolar disorder, posttraumatic stress disorder, or acute substance use disorders. Additionally, subjects with severe physical illnesses (e.g., cancer, organic brain syndromes) were excluded. The mean age at onset of the first depressive episode was 28.4 years (SD = 13.8); the mean age at initiation of first treatment was 33.5 years (SD = 14.0); and the mean age at first psychiatric hospitalization was 36.4 years (SD = 13.6). Among patients who had previously been prescribed antidepressants (*N* = 263), the mean age at first prescription was 37.8 years (SD = 12.9). A total of 292 patients (70.2%) were diagnosed with recurrent depressive disorder.

Have et al. [28] and Buckman et al. [7] demonstrated that patients with MDD and comorbid anxiety disorders tend to have a poorer prognosis. Suicidality [46] and unemployment [8] have likewise been identified as prognostically relevant factors. These characteristics should therefore be taken into account in subsequent analyses. In our sample, 92 patients (22.1%) were diagnosed with a comorbid anxiety disorder, 48 (11.5%) reported a history of suicide attempts, and 76 (18.3%) were unemployed at the time of admission.

### Treatment concept

The “Aaron T. Beck” ward follows a cognitive‒behavioral treatment approach, with a systemic and biographical understanding of the etiology and maintenance of mental disorders. The therapeutic process is based on an individualized consensus model developed collaboratively during the initial phase of treatment across several therapy sessions. This model identifies predisposing, precipitating, and maintaining factors specific to each patient. In particular, potential inhibitions of the CABS are identified and, if present, integrated into the model from the outset; most patients with MDD, for example, report a tendency to suppress or internalize anger. MDD is conceptualized as a response to acute or chronic psychosocial stressors (e.g., excessive demands at work or unemployment, relationship conflicts) in combination with individual vulnerabilities (e.g., dysfunctional beliefs, deficits in social or emotional competence, somatic problems) that lead to overload and decompensation. Treatment consists of individual sessions and group therapy and aims to support the development of effective coping mechanisms. Neurobiological or genetic hypotheses are not part of the explanatory framework. The treatment concept does not include booster sessions. After discharge, the ward provides routine medical reports to the subsequent treating providers. The overall course of treatment is illustrated in Table 1.

**Table 1:** Flowchart illustrating the overall course of treatment on the “Aaron T. Beck” ward

1. Diagnostic Phase (1–2 weeks):	This phase includes the exploration of medical and biographical history, determination of the ICD-10 diagnosis (using the IDCL), administration of questionnaires (including AHI and BDI-II), and the development of an individualized consensus model addressing etiological factors contributing to the depression and potential targets for psychotherapy. At this stage, the etiological model typically already reveals significant impairments in the CABS system. Accordingly, the CABS model is introduced through psychoeducation.
2. Intervention Phase (about 6 weeks):	Two individual psychotherapy sessions per week (with the option to involve family members) are provided, in addition to group therapies (e.g., cognitive group therapy for depression, social skills training) and adjunctive interventions (e.g., progressive muscle relaxation, occupational and work therapy, sports and physiotherapy). Inhibitions of the CABS are addressed through methods such as role-playing, facilitating emotional expression by resolving internal blocks, and practical exercises aimed at changing problematic behavior in the home environment (e.g., setting boundaries against intrusive behavior of other persons).
3. Discharge Phase (1–2 weeks):	Preliminary assessment of therapeutic progress, increased focus on transferring behavioral changes from the clinical setting to the patient’s everyday life, continued work on persisting CABS inhibitions, and planning for the period following discharge (including subsequent treatments). Administration of questionnaires.

Psychotropic medications are used as needed; however, antidepressants (ADs) are no longer part of the ward’s treatment concept (cf. [36]) and are prescribed only at the explicit request of the patients. At the time of admission, 193 patients (46.4%) were taking antidepressants. Many patients discontinued their antidepressant (AD) medication during the course of treatment. By the time of discharge, 81 patients (19.5%) were still taking antidepressants.

### Materials

The *Aggressionshemmungs-Inventar* (AHI), as described above, consists of 20 items assessing various aspects of constructive aggression. Responses are recorded on a four-point Likert scale (0 = strongly disagree, 1 = somewhat agree, 2 = mostly agree, 3 = strongly agree). Agreement levels are aggregated into a total score that quantifies the degree of inhibition of the CABS. The completion time is approximately 5–10 min. The normative sample [38] consisted of 781 healthy adults (192 men, 585 women, 4 diverse) with a mean age of 30.2 years (SD = 13.0), who were assessed with the AHI across three different studies. The AHI was administered partly in paper-and-pencil format and partly online, with no effect of the administration method. The raw AHI scores were approximately normally distributed. On average, women had higher AHI scores than men (21.2 vs. 18.5), which was accounted for when calculating z-scores. Age and educational level had no effect on AHI scores. In the current sample, Cronbach’s *α* for the AHI was 0.86 at admission and 0.91 at discharge.

The *revised Beck Depression Inventory* (BDI-II; [3]) is a widely used instrument for assessing depressive symptomatology. It includes 21 items reflecting core symptoms of MDD, each rated on a four-point scale ranging from 0 (not present) to 3 (severely expressed). The total sum score is used. Cronbach’s *α* was 0.87 at admission and 0.93 at discharge in the current sample. The BDI-II also served as the primary outcome measure for evaluating treatment effectiveness in this study.

*Sociodemographic information and medical history* were obtained through patient interviews and review of medical records.

### Procedure

Patients were asked to complete the AHI and BDI-II questionnaires upon both admission and (regularly) discharge. Six months postdischarge, patients were contacted by mail and sent the BDI-II again, accompanied by a prepaid return envelope. Readmissions for psychiatric care outside the “Aaron T. Beck” ward were ascertained through medical record review.

### Statistics

Statistical analyses were conducted using SPSS 28. Parametric correlations, one-sample, paired, and independent t-tests, the Wilcoxon signed-rank test, and hierarchical multiple regression analyses were performed (all two-tailed).

### Power analysis

A sensitivity power analysis was conducted to evaluate the statistical power associated with the current sample size (*N* = 391) for the hierarchical multiple regression analyses. Assuming a significance level of *α* = 0.05, a total of 10 predictors in the final model (including 8 control variables and 2 predictors of interest), and testing the joint incremental contribution of the 2 key predictors, the analysis indicates sufficient power to detect effects of practical relevance.

Previous findings from Study 3 by Maß and Müller-Alcazar [37] reported a substantial incremental effect of one of the predictors of interest (AHI at discharge), with *ΔR²* = 0.194, *F*(1, 233) = 114.889, *p* < .001. Power calculations performed using G*Power 3.1 [16] show that a sample size of *N* = 391 yields statistical power well above 99% to detect effects of this magnitude. Even considerably smaller effects (e.g., *ΔR²* ≈ 0.02) can still be detected with approximately 79% power. Thus, the current study is well-powered to identify both moderate and large effects, and remains adequately sensitive to detect smaller effects that have been found to be meaningful in prior research.

## Results

### General results

At admission, the mean AHI raw score was 33.9 (SD = 10.0; range = 10–56). As previous studies [37] have demonstrated that females tend to score significantly higher on the AHI than males do, raw scores were converted to standardized *z*-scores according to the manual [38] to control for gender effects. All subsequent statistical analyses involving the AHI are based on these *z*-scores. The mean AHI *z*-score at admission was 1.40 (SD = 1.01; range = -1.08–3.86). The mean BDI-II raw score was 31.6 (SD = 9.6; range = 4–58). A one-sample t-test comparing the patients’ AHI z-scores to the normative mean (0) revealed a highly significant elevation in scores (t = 28.24, df = 415, *p* < .001, 95% CI [1.31, 1.50]).

Table 2 presents the changes in AHI and BDI-II scores from admission to discharge, as analyzed using paired t-tests. Since the AHI scores were not normally distributed (admission: skewness = − 0.154, kurtosis = − 0.574; discharge: skewness = 0.378, kurtosis = − 0.280), an additional Wilcoxon signed-rank test was conducted, yielding similar results (Z = 13.603, *p* < .001).

**Table 2:** Changes in the AHI and BDI-II scores from admission to regular discharge (*N* = 391); paired t tests

	Admission x̄ (SD)	Discharge x̄ (SD)	t df = 390	*p*	d_RM_
AHI (z-scores)	1.41 (1.01)	0.40 (1.06)	17.041	< 0.001	0.880
BDI-II (total scores)	36.0 (9.5)	11.8 (9.4)	36.878	< 0.001	-1.858

AHI: Aggressionshemmungs-Inventar

BDI-II: revised Beck Depression Inventory

For 25 patients (6.0%), treatment was discontinued prematurely. Treatment dropout was defined as an unplanned or early termination of inpatient care due to a lack of treatment motivation or serious violations of ward regulations. Discharge questionnaire data were missing for these patients.

Among the 391 patients who completed treatment as scheduled, the mean duration of treatment was 58.9 days (SD = 17.9, range = 15–119).

The correlations between the AHI and BDI-II scores were *r* = .384 (*p* < .001) at admission and *r* = .569 (*p* < .001) at discharge.

### Statistical predictors of treatment outcomes

The predictive validity of the AHI was examined with respect to three different clinical outcome parameters. In this context, the CABS inhibition level at the end of treatment (i.e., the AHI score at discharge) was considered the critical predictor, rather than the change in CABS inhibition (operationalized as the pre-to-post difference in AHI scores). As described above, AHI values in the sample were, on average, substantially elevated at admission; however, a proportion of patients nevertheless presented with values within the normal range. This may be due to the fact that some patients became depressed for reasons unrelated to CABS (e.g., external stressors such as separation or job loss), which were the focus of the intervention. These patients would show normal AHI scores at both time points, resulting in a near-zero difference, which could falsely suggest treatment failure, even though they possess an intact CABS—which, according to Hypothesis 3, protects them from relapse into MDD, whether present already at admission or acquired during treatment. For control purposes, the AHI scores at admission were also included in the list of predictors.

As a *short-term treatment outcome*, the reduction in depressive symptoms during inpatient treatment was assessed by calculating the difference between BDI-II scores at admission and discharge. A second outcome parameter was the level of depressive symptoms (BDI-II) *at the 6-month follow-up.* Finally, the rate of readmission to full inpatient or day-clinic psychiatric treatment *within one year following discharge* was evaluated. This analysis focused specifically on readmissions to psychiatric facilities (including addiction medicine services) within the sector served by Klinikum Oberberg GmbH, encompassing the four locations of Marienheide, Gummersbach, Bergisch Gladbach, and Waldbröl. Readmissions to facilities outside this network could not be considered.

1. A hierarchical multiple regression analysis (HMRA) was conducted to predict *short-term treatment outcomes* among regularly discharged patients (see Table 3). The following variables were entered as predictors in the first step (Model 1): BDI-II score at admission (to control for regression to the mean; [57]), age, gender, unemployment status, history of suicide attempts, comorbid anxiety disorder, use of antidepressant medication at admission, and use of antidepressant medication at discharge. Model 1 accounted for a significant proportion of variance in short-term treatment outcomes, *R²* = 0.362, *F*(8, 382) = 267.059, *p* < .001. In the second step (Model 2), AHI (*z*-scores) at admission and discharge were added to the model, resulting in a significant increase in explained variance, *ΔR²* = 0.231, *ΔF*(2, 380) = 107.543, *p* < .001, with the total model explaining 58% of the variance (*R²* = 0.592, *F*(10, 380) = 55.231, *p* < .001). The Durbin-Watson statistic was 1.861, indicating no autocorrelation of residuals. All VIF values were below 2, indicating no problematic multicollinearity among the predictors.
2. To predict *treatment outcome at the 6-month follow-up*, a similar HMRA was conducted. A total of 296 patients (75.9% of those who were discharged regularly) participated in the follow-up assessment (see Table 4). The following variables were entered as predictors: BDI-II score at admission; BDI-II score at discharge; age; gender; unemployment status; history of suicide attempt; comorbid anxiety disorder; use of antidepressant medication at admission; use of antidepressant medication at discharge (Model 1); and AHI (*z*-scores) at admission and at discharge (Model 2). Again, model 1 accounted for a significant proportion of variance, *R²* = 0.398, *F*(9, 286) = 21.034, *p* < .001. Model 2 showed a significant increase in explained variance, *ΔR²* = 0.015, *ΔF* (2, 284) = 3.736, *p* = .025, with the total model explaining approximately 41% of the variance (*R²* = 0.414, *F*(11, 284) = 18.218, *p* < .001). The Durbin-Watson statistic was 2.139. All VIF values were below 2.
3. Finally, a third HMRA was conducted to predict psychiatric readmissions within one year after discharge (see Table 5). A total of 54 patients (13.8% of those who were discharged regularly) were readmitted during this period; the average time until readmission was 155.9 days (SD = 106.8, range = 6–364 days). In Model 1, the same set of variables was used as control variables as in the second HMRA. This model accounted for a significant proportion of variance, *R²* = 0.044, *F*(9, 381) = 1.934, *p* = .046. In Model 2, AHI (*z*-scores) at admission and discharge were added to the model, leading to a significant increase in explained variance, *ΔR²* = 0.023, *ΔF*(2, 379) = 4.736, *p* = .009, with the total model explaining about 7% of the variance in readmissions (*R²* = 0.067, *F*(11, 379) = 2.475, *p* = .005). The Durbin-Watson statistic was 2.030. All VIF values were below 2. Patients who were readmitted within one year had a mean AHI *z*-score of 1.02 (SD = 1.13) at discharge, compared with 0.30 (SD = 1.01) among those who were not readmitted (*t* = -4.790, df = 389, *p* < .001). At (first) admission, the respective AHI values were 1.64 (SD = 1.09) for readmitted patients, and 1.38 (SD = 0.099) for the not readmitted patients (*t* = -1.774, df = 389, *p* = .077).

**Table 3:** Hierarchical multiple regression analysis predicting short-term treatment outcomes with demographic and clinical variables (Model 1) and AHI *z*-scores (Model 2)

Predictor	B	SE	β	t	*p*
Model 1					
BDI-II at admission	-0.663	0.047	− 0.588	-14.150	< 0.001
age	-0.048	0.033	− 0.063	-1.451	0.148
gender	1.565	0.911	0.072	1.717	0.087
unemployment status	2.448	1.149	0.088	2.131	0.034
suicide attempts	1.012	1.415	0.030	0.716	0.475
anxiety disorder	0.196	1.083	0.007	0.181	0.857
antidepressant at admission	1.760	1.008	0.082	1.746	0.082
antidepressant at discharge	1.882	1.295	0.069	1.454	0.147
*R²*	0.362				
*ΔR²*	—				
Model 2					
BDI-II at admission	-0.687	0.041	− 0.609	-16.810	< 0.001
age	-0.049	0.027	− 0.064	-1.847	0.065
gender	1.939	0.744	0.090	2.606	0.010
unemployment status	2.446	0.923	0.088	2.649	0.008
suicide attempts	0.532	1.137	0.016	0.467	0.641
anxiety disorder	0.103	0.869	0.004	0.118	0.906
antidepressant at admission	1.097	0.810	0.051	1.355	0.176
antidepressant at discharge	-0.084	1.046	− 0.003	-0.080	0.936
AHI at admission	-2.164	0.405	− 0.204	-5.348	< 0.001
AHI at discharge	5.281	0.361	0.522	14.635	< 0.001
*R²*	0.592				
*ΔR²*	0.231				

AHI: Aggressionshemmungs-Inventar

BDI-II: revised Beck Depression Inventory

**Table 4:** Hierarchical multiple regression analysis predicting depression at 6-month follow-up with demographic and clinical variables (Model 1) and AHI *z*-scores (Model 2)

Predictor	B	SE	β	t	*p*
Model 1					
BDI-II at admission	0.358	0.063	0.282	5.661	< 0.001
BDI-II at discharge	0.589	0.069	0.443	8.600	< 0.001
age	0.105	0.042	0.120	2.495	0.013
gender	-0.434	1.176	− 0.018	-0.369	0.712
unemployment status	0.794	1.500	0.025	0.529	0.597
suicide attempts	3.580	1.847	0.090	1.939	0.054
anxiety disorder	0.571	1.342	0.020	0.425	0.671
antidepressant at admission	1.241	1.284	0.051	0.966	0.335
antidepressant at discharge	-0.972	1.624	− 0.032	-0.599	0.550
*R²*	0.398				
*ΔR²*	—				
Model 2					
BDI-II at admission	0.382	0.068	0.301	5.597	< 0.001
BDI-II at discharge	0.453	0.085	0.340	5.352	< 0.001
age	0.100	0.042	0.114	2.369	0.018
gender	0.051	1.195	0.002	0.043	0.966
unemployment status	1.280	1.497	0.040	0.855	0.393
suicide attempts	3.246	1.838	0.082	1.766	0.078
anxiety disorder	0.560	1.331	0.019	0.420	0.674
antidepressant at admission	1.149	1.273	0.047	0.903	0.368
antidepressant at discharge	-1.465	1.620	− 0.048	-0.905	0.366
AHI at admission	-0.419	0.663	− 0.034	-0.633	0.527
AHI at discharge	1.954	0.723	0.169	2.703	0.007
*R²*	0.414				
*ΔR²*	0.015				

AHI: Aggressionshemmungs-Inventar

BDI-II: revised Beck Depression Inventory

**Table 5:** Hierarchical multiple regression analysis predicting psychiatric readmissions within one year after discharge with demographic and clinical variables (Model 1) and AHI *z*-scores (Model 2)

Predictor	B	SE	β	t	*p*
Model 1					
BDI-II at admission	0.001	0.002	0.026	0.470	0.638
BDI-II at discharge	0.006	0.002	0.160	2.898	0.004
age	0.001	0.001	0.039	0.736	0.462
gender	-0.037	0.036	− 0.053	-1.031	0.303
unemployment status	-0.026	0.046	− 0.029	-0.574	0.566
suicide attempts	-0.033	0.056	− 0.030	-0.592	0.554
anxiety disorder	0.040	0.043	0.048	0.940	0.348
antidepressant at admission	0.034	0.040	0.049	0.837	0.403
antidepressant at discharge	0.046	0.051	0.052	0.886	0.376
*R²*	0.044				
*ΔR²*	—				
Model 2					
BDI-II at admission	0.001	0.002	0.024	0.412	0.680
BDI-II at discharge	0.002	0.003	0.048	0.710	0.478
age	0.001	0.001	0.036	0.691	0.490
gender	-0.016	0.037	− 0.024	-0.448	0.654
unemployment status	-0.011	0.046	− 0.012	-0.244	0.807
suicide attempts	-0.028	0.056	− 0.026	-0.505	0.614
anxiety disorder	0.036	0.043	0.043	0.853	0.394
antidepressant at admission	0.031	0.040	0.044	0.769	0.442
antidepressant at discharge	0.032	0.051	0.036	0.617	0.538
AHI at admission	0.002	0.021	0.007	0.119	0.906
AHI at discharge	0.061	0.022	0.188	2.781	0.006
*R²*	0.044				
*ΔR²*	0.023				

AHI: Aggressionshemmungs-Inventar

BDI-II: revised Beck Depression Inventory

### Analysis of dropouts

The 25 patients who discontinued treatment did not differ from those who completed treatment with respect to age, sex, AHI scores, or BDI-II scores.

Patients who did not participate in the follow-up were younger than those who did (37.4 vs. 42.0 years; *t* = -2.788, df = 389, *p* = .006). No significant differences were found with respect to sex, AHI score, or BDI-II score.

## Discussion

Inpatients with MDD exhibit pronounced inhibition of constructive aggression. Compared with normative data from previous studies in healthy individuals, the mean AHI *z*-score in our sample was 1.4, indicating that more than 90% of healthy controls had lower AHI scores. Over the course of inpatient psychotherapy, the mean AHI *z*-score of our sample decreased; at discharge, it was 0.4—still slightly above average but approaching the normative range.

Regression analyses indicate that CABS inhibition at discharge was associated with reductions in BDI-II scores during treatment, BDI-II total scores at the six-month follow-up, and readmissions within one year after discharge. Lower AHI scores at discharge were consistently associated with more favorable subsequent outcomes. Notably, this effect remained statistically significant even after adjusting for AHI scores at admission and several established prognostic factors, including depression severity during treatment, age, sex, suicidality, unemployment, and comorbid anxiety disorders. However, given the observational design of our study, these findings do not provide evidence that AHI plays a causal role in treatment response or long-term course. Consistent with our previous findings [36], no association between antidepressant medication use and outcomes was observed in the present sample. This is in line with meta-analytic evidence which suggests that combining antidepressant medication with psychotherapy does not confer substantial additional benefit compared to psychotherapy alone in the treatment of MDD [11]. Our findings suggest that CABS inhibition was associated with treatment and follow-up outcomes and may represent a psychological correlate relevant for further investigation in future longitudinal and experimental studies.

A methodological consideration concerns whether prognosis is best captured by discharge levels or by changes occurring during inpatient treatment. In the present study, we focused on CABS inhibition (operationalized by the AHI) at discharge because our sample, due to a complete census approach, comprised *all* inpatients with MDD admitted during the observation period, rather than only those with pronounced CABS inhibition. The AHI score at discharge reflects the patient’s level of CABS inhibition and may thus be relevant for relapse risk assessment. Discharge levels capture clinically meaningful between-person differences in attained recovery status, whereas change-based measures may provide complementary information regarding treatment-related processes. Future studies could examine whether changes in CABS inhibition add incremental prognostic value, for example in stratified patient groups or using alternative longitudinal modeling approaches.

Although AHI at discharge temporally precedes the follow-up period and can be used in time-lagged predictive models, it should not be conceptualized as a baseline-like risk factor. Instead, it represents a robust end-of-treatment indicator that partly reflects treatment response. Accordingly, its associations with subsequent MDD outcomes and rehospitalization risk should be interpreted as reflecting the prognostic relevance of achieved treatment response rather than suggesting temporal or causal precedence.

The introduction of a new psychological hypothesis necessitates a clear delineation from established theoretical frameworks. The following section is dedicated to this task.

### Contrasting the CABS model with other psychological concepts

*Assertiveness* is a communication style in which an individual expresses their interests or viewpoints confidently, clearly, and without fear while simultaneously respecting the rights and boundaries of others—without resorting to insult or violence. A lack of assertive behavior is often considered a consequence of social anxiety or deficits in self-esteem and social competence [53]. Assertive behavior and the CABS are likely to overlap, much like there are parallels between the suppression of anger and the inhibition of constructive aggression. However, important distinctions remain: assertiveness is viewed as an acquired social skill, whereas the CABS is considered an innate disposition, with its inhibition seen as an acquired deficit. The concept of assertiveness entails the regulation of anger, “reasonable” communication, and a cooperative orientation (e.g., willingness to compromise; [54]), with a strong emphasis on respecting others’ boundaries. In contrast, the CABS primarily serves to assert one’s own interests and needs.

*Agreeableness* is one of the dimensions of the five-factor model of personality (see [49]), characterized by altruism and helpfulness; agreeable subjects tend to be compassionate, kind, warm-hearted, trusting, cooperative, and forgiving. This trait is clearly distinct from the inhibition of the CABS, and subjects low in agreeableness—described as quarrelsome, egocentric, oppositional, competitive, and distrustful—cannot be equated with being constructively aggressive.

*Self-esteem* refers to an individual’s evaluation of their own worth, as measured, for example, by the Rosenberg Self-Esteem Scale [47]. The differences from the CABS model are evident, as self-esteem does not represent a form of behavior and is not inherently oriented toward social interaction. Nonetheless, correlations may exist; individuals with inhibited CABS may exhibit lower levels of self-esteem. Associations between self-esteem and assertiveness have also been reported [48].

*Arrested anger* refers to the tendency to suppress an emotion perceived as “wrong,” inappropriate, or threatening. Numerous studies have demonstrated that the suppression of anger can have detrimental consequences. It has been associated, for example, with hypertension [24] and coronary artery disease [13]. Furthermore, links between anger suppression and depression have also been established [22]. In some cases, suppressing anger may even lead to an increased propensity for violence [20]. Accordingly, while the suppression of anger could represent a central element of the CABS framework, the model extends beyond this to include behavioral patterns driven by other emotions as well, such as desire.

### Preliminary positioning the CABS model within core psychological frameworks

The *Frustration–Aggression Theory* [14] originally posits that frustration—the blocking of goal-directed activity—inevitably leads to destructive aggression. The theory has been modified and extended over the years, e.g., through the introduction of anger as a mediator between frustration and aggression, the distinction between instrumental and impulsive forms of aggression, and the consideration of cognitive appraisals (see [4]; for a review). Nonetheless, the findings of Frustration–Aggression Theory are not directly transferable to the CABS model, as the theory specifically addresses destructive aggression. A conceivable yet unproven link to CABS is that frustration may result from an inhibited CABS and, in turn, may lead to destructive aggression. *Transdiagnostic approaches* [12, 21] posit shared etiological roots among distinct mental disorders (e.g., anxiety and affective disorders). This perspective is motivated, on the one hand, by the high comorbidity rates observed between various disorders (e.g., MDD and panic disorder), and on the other, by the generalization of therapeutic effects to untreated comorbid conditions. Within transdiagnostic frameworks, traditional diagnostic classification systems are to be replaced by a conceptualization centered on fundamental, cross-diagnostic risk factors. For example, neuroticism [44], cognitive biases [35], and inhibited temperament [18] have been proposed as underlying mechanisms in psychopathology. The Hierarchical Taxonomy of Psychopathology (HiTOP; [33]) organizes psychopathological phenomena into a hierarchical structure based on statistical patterns of symptom co-occurrence. At the broadest level, it distinguishes several higher-order spectra (e.g., internalizing, externalizing, thought disorder, detachment), which capture shared variance across related disorders. MDD is classified as part of the internalizing spectrum, along with disorders such as anxiety disorders and post-traumatic stress disorder. Accordingly, within the HiTOP framework, inhibitions of the CABS may represent a transdiagnostic risk factor underlying disorders within the internalizing spectrum.

*Affective Neuroscience Theory* [40] posits the existence of evolutionarily ancient subcortical primary emotional systems that are similarly organized across humans and other animals. These primary emotional systems are biologically hardwired, function independently of higher cognitive processes, and form the foundation for more complex emotional and social behaviors. Seven core emotional systems have been identified: SEEKING, RAGE/ANGER, FEAR, LUST, CARE, PANIC/GRIEF, and PLAY. There is evidence that several of these systems are associated with different forms of aggression [42]. For example, the RAGE/ANGER system provides the emotional substrate for aggression in response to restraint or threat; in fight-or-flight situations, FEAR arousal can promote defensive aggression; and the PANIC/GRIEF system can be activated by social separation. Given the diversity of behavioral patterns encompassed by the CABS model, it seems plausible that CABS may be linked to multiple primary emotional systems. This hypothesis, of course, remains to be empirically tested.

The *Shared Intentionality Theory* [55] addresses the evolutionary foundations of human cooperation, which is not merely a cultural phenomenon but is rooted in deep biological mechanisms, including neural, hormonal, and genetic levels [10]. The CABS model is assumed to ultimately serve the success of social systems by promoting, for example, perceptions of fairness in the distribution of vital resources. Studies with preverbal infants suggest that certain moral evaluations, such as distinguishing between victim and aggressor, may not require learning [32]. Research on the foundations of morality in infancy has identified early-emerging sociomoral principles, including fairness and altruism, often interpreted within an evolutionary framework [50]. In a study of 18-month-old infants and 2.5-year-old toddlers, Bian et al. [5] found that children expected scarce resources to be preferentially allocated to ingroup members, whereas equal distribution between ingroup and outgroup members was expected only under resource sufficiency. These findings align with the hypothesized evolutionary origins of the CABS.

*Altruistic punishment* is a behavior observed in the Ultimatum Game [26]. In this experiment, two participants decide how to divide a sum of money. One participant, the proposer, makes an offer regarding the split, whereas the responder decides whether to accept or reject it. If the offer is accepted, both parties receive the proposed amounts; if it is rejected, neither receives anything. From a purely economic perspective, the responder should accept any nonzero offer, as this yields a better outcome than receiving nothing. However, empirical evidence consistently contradicts this prediction: across diverse cultural contexts [29], responders usually reject offers they perceive as unfair, despite incurring personal costs. The CABS model is consistent with this behavior. The ultimate cause lies in the increased likelihood that such punishment induces fairer offers in future interactions, thereby benefiting the broader social system to which both individuals belong. Studies on responder behavior in the Ultimatum Game have also included clinical samples, although findings have been inconsistent [31]. The CABS model predicts that individuals with inhibited CABS are more likely to accept offers perceived as unfair. However, empirical studies directly testing this hypothesis are lacking.

## Conclusions

The Constructive Aggression Behavioral System model offers an evolutionary theoretical framework that extends existing concepts. The present findings suggest that CABS inhibition may be clinically relevant for understanding treatment-related outcomes in MDD, potentially representing a correlate worthy of further investigation. The theoretical plausibility of the CABS model is consistent with research on infant morality and findings from behavioral economic paradigms. Basic research should evaluate the CABS model by directly examining the associations between constructive-aggressive behavior and decision-making in the ultimatum game. Clinical research should be directed toward testing the hypothesis that CABS inhibition may play a role in the development of MDD and justifying the classification of CABS inhibition within the HiTOP internalizing spectrum by demonstrating its presence across other disorders in this domain (e.g., anxiety and sexual disorders), as current clinical data are limited to patients with MDD. If future research confirms that reducing CABS inhibition is beneficial for the treatment of MDD and other mental disorders, targeted therapeutic interventions may be developed accordingly.

## Limitations

1. The naturalistic design of our observational study limits causal inference. The temporal separation between predictor and outcome variables in the regression analyses represents a necessary but not sufficient condition for such interpretations and does not support directional conclusions. Stronger evidence would require a randomized controlled design.
2. Our study does not allow any conclusions to be drawn about CABS inhibition as a risk factor for the development of MDD. This would require a longitudinal study involving individuals with no prior history of MDD. If healthy individuals exhibiting inhibited CABS show an increased risk of developing depression during the follow-up period, this would provide evidence supporting a causal relationship.
3. Although the therapeutic intervention explicitly targeted CABS inhibition (e.g., promoting boundary-setting in response to interpersonal transgressions and encouraging the expression of emotions such as anger), and improvements in depressive symptoms were observed in conjunction with reductions in CABS inhibition, it cannot be ruled out that CABS inhibition partly reflects general symptom severity or a broader process of psychological change, rather than constituting a distinct causal mechanism.
4. The CABS hypothesis inevitably shows conceptual overlap with existing, well-established constructs such as submissiveness and assertiveness. We have attempted to delineate the fundamental differences between these concepts; however, empirical evidence is still lacking at this stage. For example, head-to-head studies should compare conventional assertiveness training with training specifically designed to reduce CABS inhibition.
5. The sample consisted exclusively of inpatients from a single ward, which may limit the generalizability of the findings.
6. Self-report questionnaires such as the BDI-II may be subject to reporting biases.
7. Independent replication of our results would require a standardized treatment manual. Currently, such a manual is under development but not yet available. Interested colleagues are cordially invited to visit our unit to gain first-hand insight into our clinical procedures.
8. In the hierarchical multiple regression analyses, some key covariates were statistically controlled. Nevertheless, due to the absence of additional control variables (e.g., personality traits, therapeutic alliance, psychosocial resources) and the lack of a control group, the findings should be interpreted with caution and do not support strong or causal inferences.

## Data Availability

The datasets used and analysed during the current study are available from the corresponding author upon reasonable request.

## Abbreviations

CABS: Constructive Aggression Behavioral System
MDD: Major Depressive Disorder
AHI: Aggressionshemmungs–Inventar [Inhibited CABS Inventory]
BDI-II: Revised Beck Depression Inventory
SMRA: Stepwise multiple regression analyses
SD: Standard deviation
AD: Antidepressant
SPSS: Statistical Package for the Social Sciences
ICD-10: International Classification of Diseases, 10th Revision
